# A Target Identification Method for the Millimeter Wave Seeker via Correlation Matching and Beam Pointing

**DOI:** 10.3390/s19112530

**Published:** 2019-06-03

**Authors:** Shichao Chen, Ming Liu, Fugang Lu, Mengdao Xing

**Affiliations:** 1 No. 203 Research Institute of China Ordnance Industries, Xi’an 710065, China; chenshichao725@gmail.com (S.C.); lufugang203@163.com (F.L.); 2 School of Computer Science, Shaanxi Normal University, Xi’an 710119, China; 3 National Laboratory of Radar Signal Processing, Xidian University, Xi’an 710071, China; xmd@xidian.edu.cn

**Keywords:** target identification, correlation matching, beam pointing, millimeter wave (MMW) seeker

## Abstract

Target identification is a challenging task under land backgrounds for the millimeter wave (MMW) seeker, especially under complex backgrounds. Focusing on the problem, an effective method combining correlation matching and beam pointing is proposed in this paper. In the beginning, seeker scanning for target detection is conducted in two rounds, and target information of the detected targets is stored for correlation matching. Point or body feature judgment is implemented by using high resolution range profile (HRRP). Then, the error distribution zone is constructed with the beam pointing as the origin. In the end, we identify the target by searching the one which lies in the closest error distribution from the beam pointing center. The effectiveness of the proposed method is verified by using mooring test-fly and real flight data.

## 1. Introduction

The millimeter wave (MMW) seeker [1,2,3] is capable of working under all weather conditions, day and night, and has attracted increasing popularity. However, target identification for the MMW seeker is very difficult under land backgrounds, especially under complex backgrounds. Moreover, for the MMW seeker with strict limitations of the non-deviation flight trajectory, it is unable to image the target in two dimensionalities as synthetic aperture radar (SAR) [4,5,6]. That is to say, for small range tactical missiles, one has to realize target identification just by using the high-resolution range profile (HRRP) in the range direction [7,8,9]. As a result, the performance of target identification for the MMW seeker is not satisfying in plenty of application situations.

The key point for target identification under complex backgrounds is the false alarms caused by the clutters. How to eliminate or ease the negative influences by the clutter distractions is the precondition of successful target identification. Study of the land clutters is very complex, since it is influenced by various factors, such as geographical factors, climate factors, and radio waves, etc. Focusing on the problem, plenty of effective models have been proposed to model the distribution probability of the clutter. The essential point of these models is to generate random sequences under certain principles [10,11,12].

Fortunately, through massive data of mooring test-fly experiments and real flight experiments, we have found that the most explicit characteristics of the land clutters are unstable. Said another way, for any clutter, its point feature or body feature for the seeker detection is not certain. It may appear as a point under a certain incident angle, but the same clutter distraction may appear as a body target under another incident angle. Besides, for the same target, the target or body feature may change with different time or different weather conditions. To summarize, the clutter appears a fluctuating characteristic, or its property is quite sensitive to external conditions.

This unstable body or point feature of the clutters is helpful for target identification of the MMW seeker, since the point or body feature of the interested target is usually quite stable. Or better, the target feature is much more robust under various conditions including the backgrounds and the seeker status.

Making use of the different body/point features of the clutters and the target, a target identification method using correlation matching and beam pointing of the target is presented in this paper. Since body or point features of the clutters are not stable, we conduct two round scanning of the seeker to eliminate clutter distractions. Then, point or body feature identification is further evaluated by HRRP. In the end, error distribution zone is calculated on the basis of beam pointing. The beam pointing center will be viewed as the origin. Final target identification is realized by searching the target who lies in the closest error distribution zone of the beam pointing of the seeker. The performance of the target identification can be dramatically improved by using the proposed algorithm. The proposed method can realize satisfying target identification under complex land backgrounds, which provides further support for target tracking and precise attack for the MMW guidance missile.

## 2. Target Identification via Correlation Matching and Beam Pointing

The proposed method consists of two main steps. In the first step, we conduct scanning correlation for the targets detected in both scanning rounds. Additionally, in the second step, we compare the radial and azimuth range errors with the baseline parameters. The target that lies in the closest error zone of beam pointing of the target will be regarded as the final target identification result.

### 2.1. Correlation Matching

**Figure 1:**
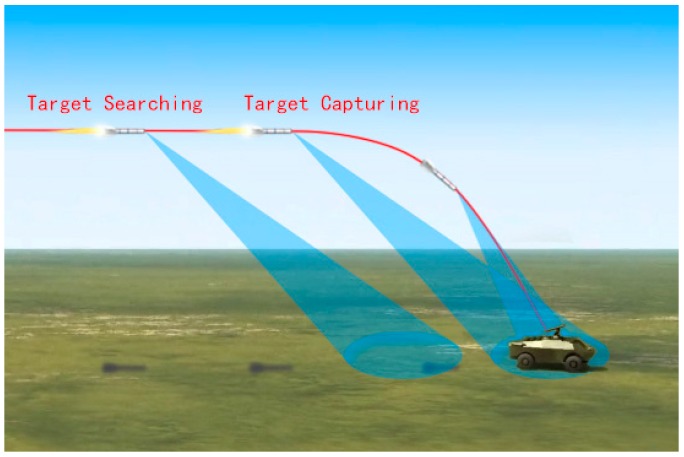
Diagram of the seeker working process.

As is known, the seeker starts to work when it reaches the effective working range, it will scan and capture the target, and output the information needed by the guidance law, such as the pitching and yawing line-of-sight (LOS) rates. The diagram of the working process of the seeker is shown in Figure 1. In the following, we will discuss the scanning correlation matching strategy of the MMW seeker in detail, which will suppress distractions effectively. Firstly, the seeker starts scanning from the left to the right by moving the gimbal angles by the servo mechanism (we name it the first round scanning in this paper), and the seeker will implement point/body feature identification based on the radar echoes, and store the information of the detected target. Then, the seeker will conduct the second round scanning, i.e., scanning from the right to the left by moving the gimbal. The same as the first round, point or body identification will also be implemented in the second round, and related target information will also be stored in the meantime for the upcoming correlation matching.

Assuming that, targets $\left(T_{1}^{1},T_{2}^{1},\ldots ,T_{i}^{1},\ldots ,T_{N_{1}}^{1}\right)$ are detected at different times $\left(t_{1}^{1},t_{2}^{1},\ldots ,t_{i}^{1},\ldots ,t_{N_{1}}^{1}\right)$ in the first round scanning by using the ordered statistic constant false alarm rate (OS-CFAR) detection [13], where $i=1,2,\ldots ,N_{1}$, $N_{1}$ represents the total number of all the detected targets. The information of seeker to target distances $\left(R_{1}^{1},R_{2}^{1},\ldots ,R_{i}^{1},\ldots ,R_{N_{1}}^{1}\right)$ and the gimbal angles $\left[\left(\vartheta_{g1}^{1},\varphi_{g1}^{1}\right),\left(\vartheta_{g2}^{1},\varphi_{g2}^{1}\right),\ldots ,\left(\vartheta_{gi}^{1},\varphi_{gi}^{1}\right),\ldots ,\left(\vartheta_{gN_{1}}^{1},\varphi_{gN_{1}}^{1}\right)\right]$ in the missile body coordinate system are stored. Similarly, interested targets $\left(T_{1}^{2},T_{2}^{2},\ldots ,T_{j}^{2},\ldots ,T_{N_{2}}^{2}\right)$ are detected at time $\left(t_{1}^{2},t_{2}^{2},\ldots ,t_{j}^{2},\ldots ,t_{N_{2}}^{2}\right)$ in the second round scanning, where $j=1,2,\ldots ,N_{2}$, $N_{2}$ is the total number of detected targets in the second round scanning. The targets are also detected by using the OS-CFAR detection just as what is implemented in the first round scanning. The corresponding information $\left(R_{1}^{2},R_{2}^{2},\ldots ,R_{j}^{2},\ldots ,R_{N_{2}}^{2}\right)$ and $\left[\left(\vartheta_{g1}^{2},\varphi_{g1}^{2}\right),\left(\vartheta_{g2}^{2},\varphi_{g2}^{2}\right),\ldots ,\left(\vartheta_{gj}^{2},\varphi_{gj}^{2}\right),\ldots ,\left(\vartheta_{gN_{2}}^{2},\varphi_{gN_{2}}^{2}\right)\right]$ associated with the seeker and the target for the second round scanning are stored for correlation matching.

Having the target information from the seeker by the two round scanning process in hand, we will carry out correlation matching in the following. However, since the target information is obtained at different times, we have to realize time synchronization in the beginning. In this paper, we will compensate the parameters obtained from the first round scanning to the second round scanning. In other words, we will update the parameters of the detected targets $\left(T_{1}^{1},T_{2}^{1},\ldots ,T_{i}^{1},\ldots ,T_{N_{1}}^{1}\right)$ in the first round scanning to expected values at moments $\left(t_{1}^{2},t_{2}^{2},\ldots ,t_{i}^{2},\ldots ,t_{N_{1}}^{2}\right)$. Then, we will compare these parameters to the ones obtained by the seeker itself in the second round scanning. If the correlation matching for the targets has been successful, we will view the successfully matched targets as the interested ones.

Here, we display the whole scanning matching process again to improve clarity. Firstly, target positions obtained in the first round scanning by the seeker in the inertial coordinate system are calculated by using coordinate system transformation. Then, target positions are updated by using the velocity information obtained from the inertial navigation system (INS), calculating target positions at the moments of target detection in the second round scanning in real time. In the following, update parameters of the seeker to target distances, the pitching gimbal angles and the yawing gimbal angles are calculated using trigonometric functions. In the end, correlation matching is implemented by making comparisons between the updated parameters and the ones outputted by the seeker itself in the second round scanning under certain restricted conditions. To further clarify the process, we will give a more detailed discussion on the assumption of only one detected target in the first round scanning.

The target position obtained in the first round scanning in the tracker coordinate system can be expressed as $\left(x_{m},y_{m},z_{m}\right)$

(1) $$\left\{ \begin{array}{l} x_{m}=R_{t_{1}}^{1}\cos\varepsilon_{m}\cos\beta_{m}\\ y_{m}=R_{t_{1}}^{1}\sin\varepsilon_{m}\\ z_{m}=-R_{t_{1}}^{1}\cos\varepsilon_{m}\sin\beta_{m} \end{array}\right.,$$

where $R_{t_{1}}^{1}$ denotes the seeker to target distance at time $t_{1}$, $\varepsilon_{m}$ denotes the misalignment angle in the pitching direction, and $\beta_{m}$ represents the misalignment angle in the yawing direction.

After coordinate transformation, target position $\left(x_{m1},y_{m1},z_{m1}\right)$ in the body coordinate system can be given by

(2) $$\left[\begin{array}{c} x_{m1}\\ y_{m1}\\ z_{m1} \end{array}\right]=A_{1}\left[\begin{array}{c} x_{m}\\ y_{m}\\ z_{m} \end{array}\right],$$

where $A_{1}$ is the direction cosine matrix.

(3) $$A_{1}=\left[\begin{array}{ccc} \begin{array}{c} \cos\vartheta_{g}\cos\varphi_{g}\\ \sin\vartheta_{g}\\ -\cos\vartheta_{g}\sin\varphi_{g} \end{array} & \begin{array}{c} -\sin\vartheta_{g}\cos\varphi_{g}\\ \cos\vartheta_{g}\\ \sin\vartheta_{g}\sin\varphi_{g} \end{array} & \begin{array}{c} \sin\varphi_{g}\\ 1\\ \cos\vartheta_{g} \end{array} \end{array}\right],$$

where $\varphi_{g}$ represents the yawing gimbal angular, and $\vartheta_{g}$ represents the pitching gimbal angular.

As previously discussed, so as to achieve scanning correlation matching, we need to realize time synchronization firstly. Said another way, we need to update the seeker parameters associated with the same target (seeker to target distance and the gimbal angles in the body coordinate system) by the time detected in the second round scanning to realize further correlation matching.

Target position $\left(x_{m2},y_{m2},z_{m2}\right)$ in the navigation coordinate system can be expressed as [14]

(4) $$\left[\begin{array}{c} x_{m2}\\ y_{m2}\\ z_{m2} \end{array}\right]=A_{2}\left[\begin{array}{c} x_{m1}\\ y_{m1}\\ z_{m1} \end{array}\right],$$

where

(5) $$A_{2}=\left[\begin{array}{ccc} \begin{array}{c} \cos\vartheta \cos\varphi \\ \sin\vartheta \\ -\cos\vartheta \sin\varphi \end{array} & \begin{array}{c} -\sin\vartheta \cos\varphi \cos\gamma +\sin\varphi \sin\gamma \\ \cos\vartheta \cos\gamma \\ \sin\vartheta \sin\varphi \cos\gamma +\cos\varphi \sin\gamma \end{array} & \begin{array}{c} \sin\vartheta \cos\varphi \sin\gamma +\sin\varphi \cos\gamma \\ -\cos\vartheta \sin\gamma \\ -\sin\vartheta \sin\varphi \sin\gamma +\cos\varphi \cos\gamma \end{array} \end{array}\right],$$

where (ϑ,φ,γ) denotes the attitude angle set, which represents the pitching angle, the yawing angle, and the rolling angle, respectively. These parameters represent the attitude of the seeker, which can be obtained by the INS.

Target position $\left(x_{m3},y_{m3},z_{m3}\right)$ can be updated in real time during the flight by

(6) $$\left\{ \begin{array}{l} x_{m3}=x_{m2}-\int_{t_{1}}^{t^{\prime \prime }}v_{x}\left(t\right)dt\\ y_{m3}=y_{m2}-\int_{t_{1}}^{t^{\prime \prime }}v_{y}\left(t\right)dt\\ z_{m3}=z_{m2}-\int_{t_{1}}^{t^{\prime \prime }}v_{z}\left(t\right)dt \end{array}\right.,$$

where $v_{x}\left(t\right)$, $v_{y}\left(t\right)$, and $v_{z}\left(t\right)$ represents the north velocity, vertical velocity, and east velocity of the seeker, respectively. $t^{\prime \prime }$ is the time when the seeker detects the target in the second round scanning. The scanning speed is very fast, and the scanning angular scope is limited. Therefore, the time interval $\Delta t=t^{\prime \prime }-t_{1}$ is usually quite small. The variance of the speed vector of the seeker can be neglected, and Equation (6) can be simplified by

(7) $$\left\{ \begin{array}{l} x_{m3}=x_{m2}-v_{x}\Delta t\\ y_{m3}=y_{m2}-v_{y}\Delta t\\ z_{m3}=z_{m2}-v_{z}\Delta t \end{array}\right..$$

So far, we have obtained the updated target position at time $t^{\prime \prime }$. Or better, $\left(x_{m3},y_{m3},z_{m3}\right)$ can be viewed as the compensated result for the first round scanning. What follows is to calculate the pitching and yawing gimbal angles. The target position $\left(x_{m4},y_{m4},z_{m4}\right)$ in the body coordinate system can be expressed as

(8) $$\left[\begin{array}{c} x_{m4}\\ y_{m4}\\ z_{m4} \end{array}\right]=A_{2}^{T}\left[\begin{array}{c} x_{m3}\\ y_{m3}\\ z_{m3} \end{array}\right].$$

Seeker parameters associated with the target can be calculated by using $\left(x_{m4},y_{m4},z_{m4}\right)$. The seeker to target distance can be given by

(9) $$R^{\prime }=\sqrt{x_{m4}^{2}+y_{m4}^{2}+z_{m4}^{2}}.$$

The beam pointing angle of the target along the pitching direction $\vartheta_{g}^{\prime }$ can be given by

(10) $$\vartheta_{g}^{\prime }=arc\sin\left(y_{m4}/\sqrt{x_{m4}^{2}+y_{m4}^{2}+z_{m4}^{2}}\right).$$

Additionally, the beam pointing angle of the target along the yawing direction $\varphi_{g}^{\prime }$ can be given by

(11) $$\varphi_{g}^{\prime }=arc\tan\left(z_{m4}/x_{m4}\right).$$

Correlation matching is implemented by comparing the parameters of $R^{\prime }$, $\vartheta_{g}^{\prime }$ and $\varphi_{g}^{\prime }$ at time $t^{\prime \prime }$ with the corresponding parameters outputted by the seeker at this time.

(12) $$\left\{ \begin{array}{l} |R^{\prime }-R|\leq \Delta R\\ |\vartheta_{g}^{\prime }-\vartheta_{g}|\leq \Delta \vartheta_{g}\\ |\varphi_{g}^{\prime }-\varphi_{g}|\leq \Delta \varphi_{g} \end{array}\right.,$$

where R is the seeker to target distance, $\vartheta_{g}$ is the gimbal angle in the pitching direction, and $\varphi_{g}$ is the gimbal angle in the yawing direction outputted by the seeker itself at this time, ΔR, $\Delta \vartheta_{g}$, and $\Delta \varphi_{g}$ are the thresholds for correlation matching.

We have to note that these values are quite different under different circumstances. We have to determine them according to the real system in practice. For different INS equipment, the measurement precision of $v_{x}$, $v_{y}$, and $v_{z}$ are different, leading to different beam pointing directions. Moreover, the error of the gimbal angle output and the angle measurement error are also inevitable. Briefly, the determination of the parameters of ΔR, $\Delta \vartheta_{g}$, and $\Delta \varphi_{g}$ greatly relies on real application conditions, which have to be adjusted in practice.

### 2.2. Error Calculation of Radial Range and Azimuth Range

For complex land backgrounds, after correlation matching between two round scanning, multiple targets may still meet the restriction of correlation matching. We cannot realize target identification just by using the proposed method presented in Section 2.1. Assuming that, there are K targets $\left\{T_{c}^{1},T_{c}^{2},\ldots ,T_{c}^{K}\right\}$ which satisfy the condition restricted by Equation (12), the corresponding parameters of the seeker are $\left[\left(\vartheta_{gc1},\varphi_{gc1}\right),\left(\vartheta_{gc2},\varphi_{gc2}\right),\ldots ,\left(\vartheta_{gcK},\varphi_{gcK}\right)\right]$ and $\left(R_{c1},R_{c2},\ldots ,R_{cK}\right)$, respectively. In the following, we calculate the radial range error and the azimuth range error. Target position $\left(x_{t},y_{t},z_{t}\right)$ calculated in the navigation coordinate system can be expressed as

(13) $$\begin{array}{l} x_{t}=R\cdot \cos\left(\theta_{n0}\right)\cdot \cos\left(\varphi_{n0}\right)\\ y_{t}=R\cdot \sin\left(\theta_{n0}\right)\\ z_{t}=R\cdot \cos\left(\theta_{n0}\right)\cdot \sin\left(\varphi_{n0}\right) \end{array}\},$$

where R represents the seeker to target position calculated by the fire-control system, $\theta_{n0}$ is the pitching angle, and $\varphi_{n0}$ is the azimuth angle. 

Additionally, target position $\left(x_{t0},y_{t0},z_{t0}\right)$ in the launching system can be given by

(14) $$\left[\begin{array}{c} x_{t0}\\ y_{t0}\\ z_{t0} \end{array}\right]=A\left(\varphi_{bx}\right)\left[\begin{array}{c} x_{t}\\ y_{t}\\ z_{t} \end{array}\right],$$

where $\varphi_{bx}$ represents the north angle, the direction cosine matrix A can be given by

(15) $$A\left(\varphi_{bx}\right)=\left[\begin{array}{ccc} \begin{array}{c} \cos\varphi_{bx}\\ 0\\ \sin\varphi_{bx} \end{array} & \begin{array}{c} 0\\ 1\\ 0 \end{array} & \begin{array}{c} -\sin\varphi_{bx}\\ 0\\ \cos\varphi_{bx} \end{array} \end{array}\right].$$

Since the position $\left(x_{m},y_{m},z_{m}\right)$ can be obtained by the INS mounted on the seeker, the seeker to target distance $R^{\prime \prime }$ can be calculated in real time

(16) $$R^{\prime \prime }=\sqrt{{\left(x_{t0}-x_{m}\right)}^{2}+{\left(y_{t0}-y_{m}\right)}^{2}+{\left(z_{t0}-z_{m}\right)}^{2}}.$$

For the kth (k=1,2,…,K) target, the radial range error $\Delta R_{ck}^{r}$ can be given by

(17) $$\Delta R_{ck}^{r}=R_{ck}-R^{\prime \prime }.$$

According to the geometry relationship between the seeker and the target, the beam pointing direction of the yaw angle $\varphi_{pt}$ can be expressed as

(18) $$\varphi_{pt}=arc\tan\left(\frac{z_{t0}-z_{m}}{x_{t0}-x_{m}}\right).$$

Additionally, the azimuth range error can be given by

(19) $$\Delta R_{ck}^{a}=R_{ck}\cdot \left(\varphi_{gcK}-\varphi_{pt}\right).$$

**Figure 2:**
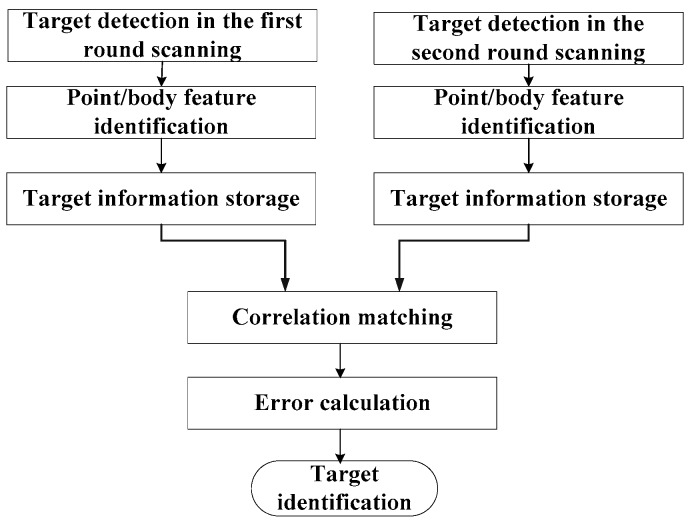
The flow diagram of the proposed algorithm.

Hereto, we obtained the range errors of the successfully matched K targets $\left\{\left(\Delta R_{ck}^{r},\Delta R_{ck}^{a}\right),\left(\Delta R_{ck}^{r},\Delta R_{ck}^{a}\right),\ldots ,\left(\Delta R_{ck}^{r},\Delta R_{ck}^{a}\right),\ldots ,\left(\Delta R_{cK}^{r},\Delta R_{cK}^{a}\right)\right\}$. What follows is to calculate the location of the targets at the error distribution zone. The area was divided into 1σ, 2σ, and 3σ error distribution zones empirically according to massive mooring test-fly data and real flight data exploration with the consideration of various error introduction terms. Final target identification is conducted by locating the target, which lies in the closest error distribution zone with respect to the beam pointing of the seeker. The flow diagram of the proposed method is displayed in Figure 2.

## 3. Experimental Results and Analysis

Mooring test-fly data and real flight data were used to verify the effectiveness of the proposed method.

### 3.1. Mooring Test-Fly Experiment

Firstly, we tested the proposed method on a mooring test-fly experiment. Here, we will give an explanation of the mooring test-fly experiment. As is known, a seeker is very expensive, especially for the MMW seeker. As a result, before a seeker is mounted on the missile for launching, we have to evaluate its performance carefully. To fulfill the task, we usually conduct the mooring test-fly experiment. In the mooring test-fly experiment, we mounted the seeker on a platform, such as a helicopter with pilots or an unmanned aerial vehicle (UAV) with remote control on the ground. The platform with the seeker mounted on will move according to the missile trajectory. When the distance from the seeker to the target is within the effective range of the seeker, the seeker will start to illuminate an electromagnetic wave, and implement all the processing just as the real missile fight case. The track will continue until the platform approaches the target, i.e., the distance from the seeker to the target is zero. Then, the platform will fly high and far from the target, getting ready for the next round trip for seeker data collection and performance evaluation. Since the seeker still exists, we can conduct similar processes again and again to search satisfying parameters. For the MMW seeker that we focus on, the platform with the MMW seeker mounted on flies a simulated trajectory. The thresholds for correlation matching are set to be 15 m for range, and 2° for angles, respectively.

The seeker starts to work when reaching the effective working range. Two round scanning of the seeker was conducted subsequently. A total of 11 targets were detected in the first round scanning. The corresponding target information is tabulated in Table 1, target 1-c (c=1,2,…11) denotes the cth detected target. We can know that target 1-3 is the accurate target by the prior information calculation from target location and seeker position obtained by the high-precision global position system (GPS) mounted on the target and the INS mounted on the seeker [15,16].

**Figure 3:**
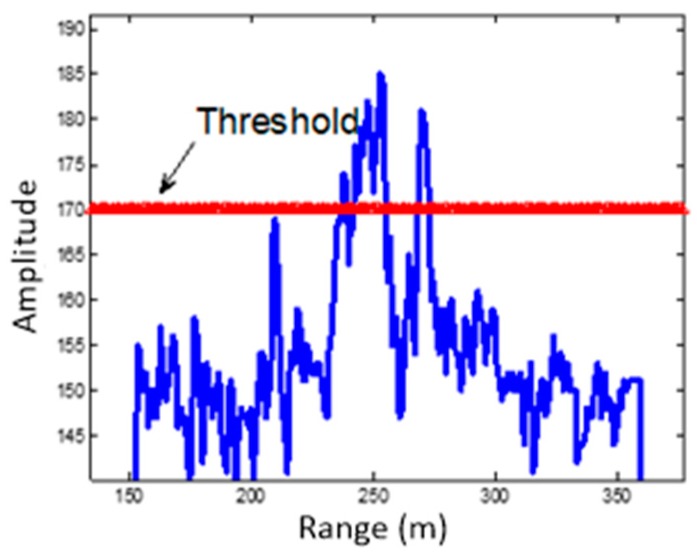
High resolution range profile (HRRP) of a detected target.

**Figure 4:**
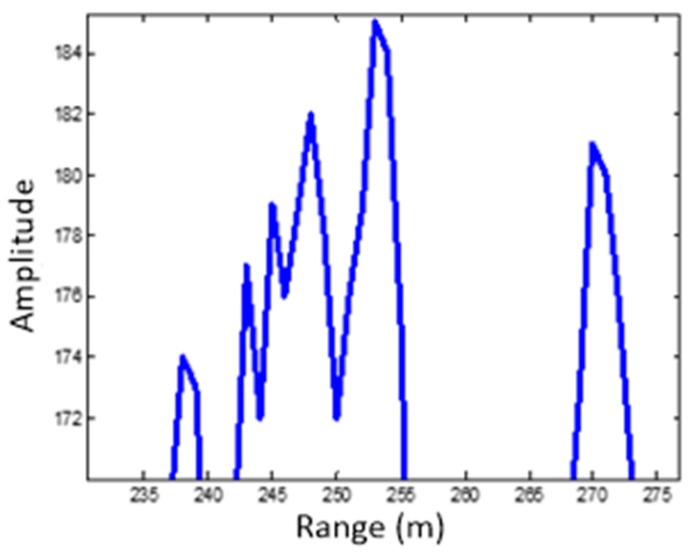
HRRP after target detection.

As previously discussed, point/body feature of the target can be determined by using the HRRP [7,8,9]. The HRRP of the MMW echo is given in Figure 3. Additionally, the profile after target detection is shown in Figure 4. As can be seen, there are two objects that exceed the threshold. One appears as a body target, which coincides with the size of a tank, and the other one appears as a point target.

As can be seen in Table 1, targets that appear as point features will be discarded (targets 1-5 and 1-7 in Table 1), and the five strongest reflection targets with body features will be chosen for correlation matching, i.e., targets 1-1, 1-2, 1-3, 1-4, and 1-6.

Then, the seeker implemented the second round scanning, and 10 targets were detected in this round scanning. The corresponding target information is shown in Table 2. Similar to Table 1, 2-n (n=1,2,…10) denotes the nth target in the second round scanning.

Likewise, discarding the targets appearing as point features (targets 2-7, 2-8, and 2-10 in Table 2), and choosing the five strongest reflection targets with body features, we can get targets 2-1, 2-2, 2-3, 2-4 and 2-5 for correlation matching.

Correlation matching results for targets 1-1, 1-2, 1-3, 1-4, and 1-6 are given in Table 3, Table 4, Table 5, Table 6 and Table 7, respectively.

From Table 3, we can see that only target 2-1 can realize satisfying matching with target 1-1. The range difference is only 6 m, and the value is the smallest of all the candidate targets. We can tell that the range difference may be viewed as the top choice for correlation matching. In addition, the second effective factor is the pitching angle difference. The azimuth angle can provide further assistance.

From Table 4, we can see that only target 2-2 realizes satisfying matching with target 1-2, and we can further find that the range difference of target 2-4 is also quite small, which is 24 m. Although the range difference is the top choice, we have to set the threshold appropriately to avoid misjudging. Combining the pitching and azimuth angles provides great discriminating power.

From Table 5, we can see that target 2-3 realizes satisfying matching with target 1-3. In addition, from Table 6, we can see that there is no target that matches target 1-4. All the targets selected in the second round scanning cannot match target 1-4. In other words, target 1-4 will not arrive at the final stage of target identification. From Table 7, we can see that target 1-6 realizes satisfying correlation with target 2-4.

**Figure 5:**
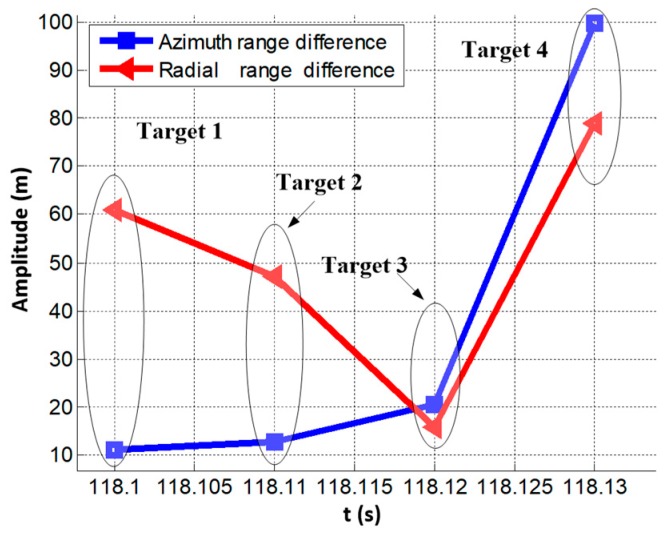
Radial and azimuth range differences.

To summarize, after two round target correlation matching, the following target pairs realize satisfying matching: targets 1-1 and 2-1, targets 1-2 and 2-2, targets 1-3 and 2-3, targets 1-6 and 2-4. Here, we label the successfully matched targets as target 1, target 2, target 3, and target 4, respectively. The radial range and azimuth range errors with respect to the beam pointing center are given in Figure 5. In Figure 5, the blue line and the red line represent the distance differences in radial and azimuth directions with respect to the origin, i.e., the beam pointing center, respectively.

Then, we calculated error distances associated with the error distribution zone. Just as the determination of the thresholds set for correlation matching, the calculation of the error distribution zone is even more complex. The determination of the parameters along the range and azimuth directions involves plenty of error terms, such as target orientation error by the fire control system, floating error of the INS, and angle determination error of the seeker, etc. Here, we set the parameters empirically through massive data exploration again. The 1σ error distribution rectangular zone is formed by 35 and 50 m in the radial and azimuth directions, respectively. Said another way, for a given seeker, the determination of these parameters is achieved by excessive times of mooring test-fly experiments to support the real missile flight.

**Figure 6:**
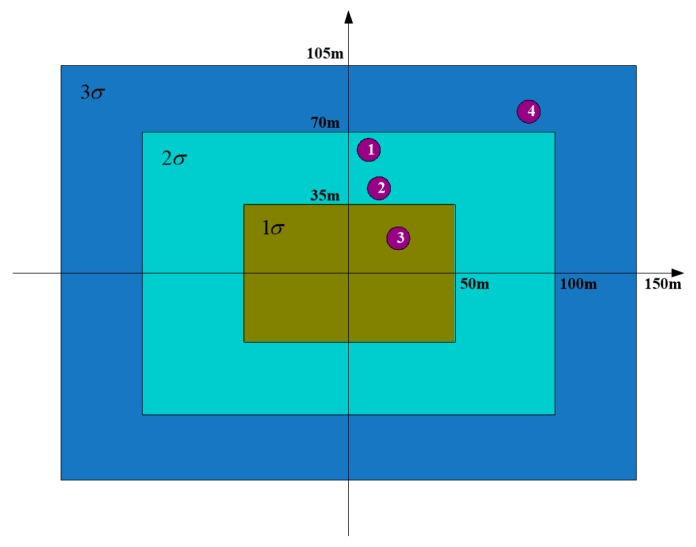
Error distribution zone of the interested targets.

According to the geometry between the interested targets and the beam pointing center, the error distribution zone with the four successfully matched targets are shown in Figure 6. The abscissa denotes the azimuth range error, and the ordinate denotes the radial range error. From Figure 6, we can see that target 1 and target 2 lie in the 2σ error zone of the beam pointing center. Target 4 lies in the 3σ error zone of the beam pointing center. Only target 3 lies in the 1σ error zone of the beam pointing center. From Figure 6, we can see that target 3 lies in the closest error distribution zone of the beam pointing of the seeker. Target 3 will be viewed as the interested target for the seeker. We can see that target identification can be obtained, and the proposed method is capable of providing powerful assistance and support for precise attack of the missile.

Here, we will give more explanations of the parameters, which will affect the final identification result. Although satisfying results can be obtained by using the proposed method, parameter setting is of great importance, and appropriate parameter setting is the precondition of accurate identification. When the parameters are not well-selected, wrong target identification will emerge. In this part, we will display examples in situations with inappropriate parameter setting. Firstly, we take the azimuth angle difference threshold as an example. From Table 3, we can see that if we set the azimuth angle difference threshold to be less than 1.48°, the correlation matching for the interested target 3 will fail. In other words, target 3 will not be selected for further processing. Wrong identification will inevitably occur.

**Figure 7:**
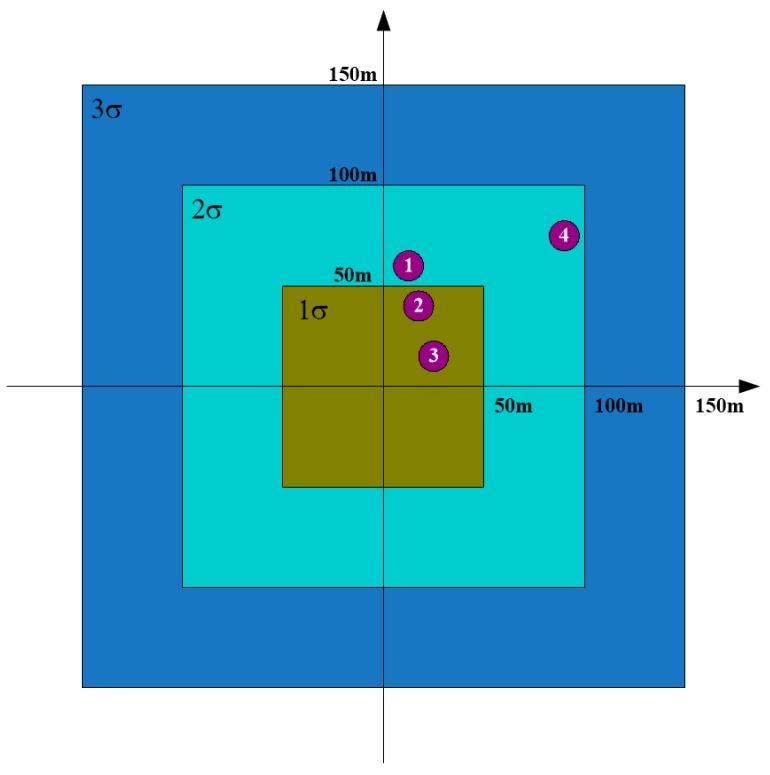
Error distribution zone of the interested targets with radial range threshold of 50 m.

In the following, we take the error distribution zone threshold as another example of inappropriate parameter setting for illustration. In the experiment, the 1σ error distribution zone is given by the inside rectangular with radial range threshold of 35 m and azimuth range threshold of 50 m, as shown in Figure 6. If we set the radial range threshold to be 50 m, the error distribution zone for the targets will change, as shown in Figure 7. Comparing Figure 6 with Figure 7, we can see that for the four interested targets, although target 1 and target 3 still lie in the same error distribution zone with different radial range thresholds, target 2 and target 4 change their error distribution zones. Target 2 lies in the 2σ error distribution zone in Figure 6, whereas it lies in the 1σ error distribution zone in Figure 7. As for target 4, it lies in the 3σ error distribution zone in Figure 6, but it approaches to the 2σ error distribution zone in Figure 7.

As can be seen, both target 2 and target 3 lie in the 1σ error distribution zone in Figure 7. We will choose the target with larger amplitude to be the final identification result, when more than one target lies in the same error distribution zone. That is to say, wrong target identification will emerge in this situation. Target 2 corresponds to target 1-2 in the first round and target 2-2 in the second round, whereas target 3 corresponds to target 1-3 in the first round and target 2-3 in the second round. From Table 1, we can see that the amplitude of target 1-2 is 120, and the amplitude of target 1-3 is 100. From Table 2, we can see that the amplitude of target 2-2 is 120, whereas the amplitude of target 2-3 is 111. As can be seen, the amplitude of target 2 is larger than that of target 3 in both rounds. In other words, target 2 will be viewed to be more threatening than target 3. This is definitely what we would like to avoid.

Above all, appropriate parameter setting is of great importance to target identification by using the proposed method. Inappropriate parameter setting will lead to undesirable failure. That is to say, one has to adjust the parameters according to the real seeker system in practice.

### 3.2. Real Data

In the following, we verify the effectiveness of the proposed method by using real flight data of the seeker. The background of the targets is not as complex as the one in mooring test-fly experiment. A total of 14 targets were detected in the first round scanning, and the target information is shown in Table 8. In the experiment, target 1-1 is the interested target. Fifteen targets were detected in the second round scanning, and the corresponding target information is shown in Table 9. Firstly, we will select the five largest targets with body features as correlation matching candidates just as the case in Section 3.1. That is to say, we would like to implement correlation matching between target sets of 1-1, 1-2, 1-3, 1-4 1-5 detected in the first round scanning and target sets of 2-1, 2-2, 2-3, 2-4, and 2-5 in the second round scanning. The correlation matching results for targets 1-1, 1-2, 1-3, 1-4, and 1-5 are shown in Table 10, Table 11, Table 12, Table 13 and Table 14, respectively.

From the experimental results we can tell that all the targets fail to realize successful correlation matching except target 1-1. We can see that from Table 10, target 1-1 realizes satisfying matching with target 2-2. The seeker will view target 1-1 as the interested target to attack. The radial and azimuth range errors are 50 and 116.7 m, respectively. The thresholds for error distribution zone are set to be the same as the mooring test-fly experiment (actually, as previously discussed, the parameters are determined by excessive mooring test-fly experiments). In other words, the target lies in the 3σ error zone of the beam pointing center. Real flight data further verifies the effectiveness of the proposed method.

## 4. Conclusions

So as to overcome the great obstacle of target identification in land backgrounds for the MMW seeker, especially under complex conditions, an effective method taking the unstable body or point feature characteristic of the clutters into account is presented in this paper. Clutter distractions are discarded effectively through information correlation matching by using two round scanning of the seeker. A satisfying target identification result is obtained by combining the prior beam pointing information.

Moreover, appropriate parameter setting of the thresholds as well as the appropriate construction of the error distribution zone is the precondition of the proposed method. However, the determination of the parameters is restricted by various factors. The parameters of the proposed method presented in this paper is determined by the exploration and summation of plenty of real data including mooring test-fly and real flight experimental data. How to construct a precise model for the determination of these parameters deserves further study.

## Figures and Tables

**Table 1:** Detected target information in the first round scanning.

Target	1-1	1-2	1-3	1-4	1-5	1-6	1-7	1-8	1-9	1-10	1-11
Point/Body	body	body	body	body	point	body	point	body	body	body	body
Amplitude	121	120	111	106	105	102	101	100	94	92	92
Pitching angle (°)	1.17	1.11	1.14	1.25	1.23	1.18	1.18	1.15	1.14	1.29	1.31
Azimuth angle (°)	−3.24	−3.71	−4.71	−0.57	0.77	−7.25	−7.26	−0.93	−6.66	1.49	1.01
Seeker to target distance (m)	1695	1806	1733	1754	1695	1837	1894	1652	1642	1896	1839

**Table 2:** Detected target information in the second round scanning.

Target	2-1	2-2	2-3	2-4	2-5	2-6	2-7	2-8	2-9	2-10
Point/Body	body	body	body	body	body	point	point	point	body	point
Amplitude	121	120	112	103	102	102	99	99	93	78
Pitching angle (°)	1.25	1.14	1.2	1.19	1.18	1.43	1.43	1.28	1.17	1.19
Azimuth angle (°)	−2.14	−2.97	−3.23	−5.9	−0.76	−6.68	−6.32	−5.71	1.9	2.85
Seeker to target distance (m)	1689	1798	1735	1830	1644	1887	1689	1635	1886	1797

**Table 3:** Correlation matching results for target 1-1.

Target	2-1	2-2	2-3	2-4	2-5	2-9
Pitching angle difference (°)	0.08	0.03	0.03	0.02	0.01	0
Azimuth angle difference (°)	1.1	0.27	0.01	2.66	2.48	5.14
Range difference (m)	6	103	40	135	51	191

**Table 4:** Correlation matching results for target 1-2.

Target	2-1	2-2	2-3	2-4	2-5	2-9
Pitching angle difference (°)	0.14	0.03	0.09	0.08	0.07	0.06
Azimuth angle difference (°)	1.57	0.74	0.48	2.19	2.95	5.61
Range difference (m)	117	8	71	24	162	80

**Table 5:** Correlation matching results for target 1-3.

Target	2-1	2-2	2-3	2-4	2-5	2-9
Pitching angle difference (°)	0.11	0	0.06	0.05	0.04	0.03
Azimuth angle difference (°)	2.57	1.74	1.48	1.19	3.95	6.61
Range difference (m)	44	65	2	97	89	153

**Table 6:** Correlation matching results for target 1-4.

Target	2-1	2-2	2-3	2-4	2-5	2-9
Pitching angle difference (°)	0	0.11	0.05	0.06	0.07	0.08
Azimuth angle difference (°)	1.57	2.4	2.66	5.33	0.19	2.47
Range difference (m)	65	44	19	76	110	132

**Table 7:** Correlation matching results for target 1-6.

Target	2-1	2-2	2-3	2-4	2-5	2-9
Pitching angle difference (°)	0.07	0.04	0.02	0.01	0.00	0.01
Azimuth angle difference (°)	5.11	4.28	4.02	1.35	6.49	9.15
Range difference (m)	148	39	102	7	193	49

**Table 8:** Detected target information in the first round scanning for real flight data.

Target	1-1	1-2	1-3	1-4	1-5	1-6	1-7	1-8	1-9	1-10	1-11	1-12	1-13	1-14
Point/Body	body	body	body	body	body	body	body	body	point	point	body	body	point	point
Amplitude	110	96	95	95	95	94	94	94	93	92	92	91	90	90
Pitching angle (°)	−6.98	−7.01	−7.05	−7.04	−7.04	−7.1	−7.09	−7.01	−7.05	−7.04	−6.96	−6.97	−7.1	−7.1
Azimuth angle (°)	−1.01	1.17	2.62	3.91	3.91	4.12	−5.12	1.17	2.62	−4.47	−0.77	−2.31	−5.21	−5.21
Seeker to target distance (m)	2298	2163	2206	2337	2304	2406	2205	2262	2430	2437	2363	2257	2235	2364

**Table 9:** Detected target information in the second round scanning for real flight data.

Target	2-1	2-2	2-3	2-4	2-5	2-6	2-7	2-8	2-9	2-10	2-11	2-12	2-13	2-14	2-15
Point/Body	body	body	body	body	body	body	body	body	body	body	point	body	body	point	body
Amplitude	107	98	97	96	96	95	95	95	95	95	94	94	94	94	94
Pitching angle (°)	−6.99	−7	−7	−7.1	−7.09	−7	−7.09	−7.04	−7.01	−7.01	−6.88	−7.03	−7	−6.97	−6.99
Azimuth angle (°)	1.32	−2.73	−0.26	4.55	5.22	2.33	5.22	3.5	0.39	1.11	−3.81	−2.17	0.9	−3.61	−3.38
Seeker to target distance (m)	2294	2295	2185	2441	2261	2150	2303	2341	2230	2395	2343	2153	2261	2411	2380

**Table 10:** Correlation matching results for target 1-1 for real flight data.

Target	2-1	2-2	2-3	2-4	2-5	2-9
Pitching angle difference (°)	0.01	0.02	0.02	0.02	0.12	0.11
Azimuth angle difference (°)	2.33	1.72	0.75	2.66	5.56	6.23
Range difference (m)	4	3	113	143	37	4

**Table 11:** Correlation matching results for target 1-2 for real flight data.

Target	2-1	2-2	2-3	2-4	2-5
Pitching angle difference (°)	0.02	0.01	0.01	0.09	0.08
Azimuth angle difference (°)	0.15	3.9	1.43	3.38	4.05
Range difference (m)	131	132	22	278	98

**Table 12:** Correlation matching results for target 1-3 for real flight data.

Target	2-1	2-2	2-3	2-4	2-5
Pitching angle difference (°)	0.06	0.05	0.05	0.05	0.04
Azimuth angle difference (°)	1.3	5.35	2.88	1.93	2.6
Range difference (m)	88	89	110	235	55

**Table 13:** Correlation matching results for target 1-4 for real flight data.

Target	2-1	2-2	2-3	2-4	2-5
Pitching angle difference (°)	0.05	0.04	0.04	0.06	0.05
Azimuth angle difference (°)	2.59	6.64	4.17	0.64	1.31
Range difference (m)	43	42	152	104	76

**Table 14:** Correlation matching results for target 1-5 for real flight data.

Target	2-1	2-2	2-3	2-4	2-5
Pitching angle difference (°)	0.05	0.04	0.04	0.06	0.05
Azimuth angle difference (°)	2.59	6.64	4.17	0.64	1.31
Range difference (m)	10	9	119	137	43
